# Atomic-scale intermolecular interaction of hydrogen with a single VOPc molecule on the Au(111) surface†

**DOI:** 10.1039/d0ra08951f

**Published:** 2021-02-03

**Authors:** Jinoh Jung, Shinjae Nam, Christoph Wolf, Andreas J. Heinrich, Jungseok Chae

**Affiliations:** Department of Physics, KAIST Daejeon 34141 Korea; Center for Quantum Nanoscience, Institute for Basic Science (IBS) Seoul 03760 Korea heinrich.andreas@qns.science chae.jungseok@qns.science; Physics Department, Ewha Womans University Seoul 03760 Korea; Ewha Womans University Seoul 03760 Korea

## Abstract

Molecular dynamics of hydrogen molecules (H_2_) on surfaces and their interactions with other molecules have been studied with the goal of improvement of hydrogen storage devices for energy applications. Recently, the dynamic behavior of a H_2_ at low temperature has been utilized in scanning tunnelling microscopy (STM) for sub-atomic resolution imaging within a single molecule. In this work, we have investigated the intermolecular interaction between H_2_ and individual vanadyl phthalocyanine (VOPc) molecules on Au(111) substrates by using STM and non-contact atomic force microscopy (NC-AFM). We measured tunnelling spectra and random telegraphic noise (RTN) on VOPc molecules to reveal the origin of the dynamic behavior of the H_2_. The tunnelling spectra show switching between two states with different tunnelling conductance as a function of sample bias voltage and RTN is measured near transition voltage between the two states. The spatial variation of the RTN indicates that the two-state fluctuation is dependent on the atomic-scale interaction of H_2_ with the VOPc molecule. Density functional theory calculations show that a H_2_ molecule can be trapped by a combination of a tip-induced electrostatic potential well and the potential formed by a VOPc underneath. We suggest the origin of the two-state noise as transition of H_2_ between minima in these potentials with barrier height of 20–30 meV. In addition, the bias dependent AFM images verify that H_2_ can be trapped and released at the tip–sample junction.

## Abstract 2

Molecular dynamics of hydrogen molecules trapped in micro-cavity between atomic-scale tip and VOPc molecules on surface and intermolecular interactions have been studied by using scanning tunneling spectroscopy and atomic force microscopy at atomic-scale.

## Graphical abstract

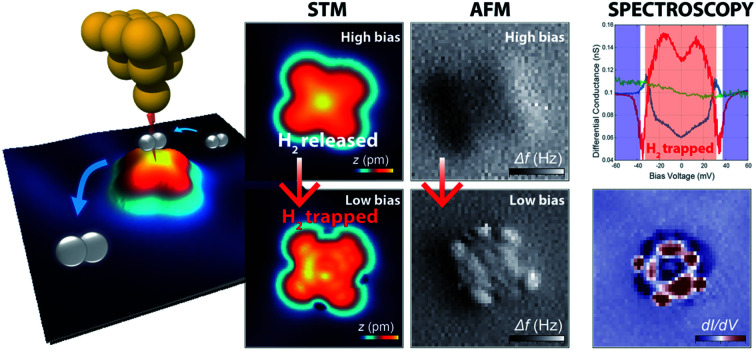

## Introduction

Hydrogen, the most abundant element in the universe, has been studied as a sustainable energy source for a long time.^1–3^ However, the understanding of the interaction of hydrogen with organic molecules at the atomic scale was limited. The study of hydrogen molecules, the lightest molecule in nature, is an experimental challenge, as it is hard to localize on surfaces where it is mobile by diffusion even at cryogenic temperatures. This makes imaging of a single hydrogen molecule very difficult for techniques capable of atomic resolution. Only recently, the stochastic behavior of hydrogen molecules on surfaces has been measured by using scanning tunneling microscopy (STM) and non-contact atomic force microscopy (NC-AFM), where it manifests as unconventional tunneling spectra with random telegraphic noise (RTN).^4,5^ The random switching of hydrogen caused by their stochastic motion in the tunnelling junction results in the driving of a macroscopic oscillator at low temperature.^4^ Conventionally, a two-state model has been suggested to explain two states that lead to fluctuations in the tunnelling conductance,^4–7^ however, the exact origin of these microstates is often an open question. Recently, a tip functionalized by a hydrogen molecule was used to perform molecular imaging with ultra-high resolution, including the mapping of chemical bonds.^8–11^ Despite several studies about ultra-high resolution imaging using H_2_ functionalized tips or H_2_ rotational excitation measurements using inelastic tunnelling spectroscopy,^12–15^ the current understanding of the intermolecular interaction of hydrogen at the atomic scale as seen by STM and NC-AFM is still incomplete.

Here, we observed the atomic-scale intermolecular interactions of H_2_ with VOPc molecules deposited on an Au(111) substrate using STM and NC-AFM. In this system, H_2_ can be trapped by the local electrostatic potential well formed by a combination of the tip-induced potential and the interaction of H_2_ with the VOPc molecule. We found that the motion of trapped H_2_ is excited when the applied bias voltage exceeds a threshold voltage. Below this threshold voltage, H_2_ is trapped between the tip and the VOPc molecule. Near the threshold voltage, the stochastic hopping between two local energy minima manifests itself as random telegraph noise in the tunnelling current. When the bias voltage far exceeds the transition voltage, H_2_ is ejected from the STM tunnelling junction by overcoming the confining potential barrier. As a result, the bias spectroscopy shows unconventional tunnelling spectra near 20–30 mV above and below the Fermi energy, which are induced by two-state fluctuation in the tunnelling conductance. AFM measurements show the repulsive character below the threshold voltage of the interaction between tip and trapped H_2_ but show only Van der Waals attractive interaction above the threshold voltage at the same tip–sample distance, which is consistent with STM measurements and density functional theory (DFT) calculations of the potential energy landscape of this system.

## Results and discussion

To prepare the sample, a clean Au(111) surface with herringbone reconstructions was obtained by several cycles of Ar sputtering and annealing with *T* = 450 °C followed by deposition of VOPc molecules at room temperature in the vacuum chamber. VOPc molecules purchased from Sigma-Aldrich (dye content, >90%) were used as it delivered and deposited on the substrate by the effusion cell evaporator with *T* = 400 °C. Before deposition, the evaporator was degassed overnight at 300–350 °C in the vacuum chamber. On the cooled down sample in *T* = 8.5 K, the hydrogen molecules (∼10^−9^ Torr of the partial pressure) were naturally accumulated with several days.

**Fig. 1:**
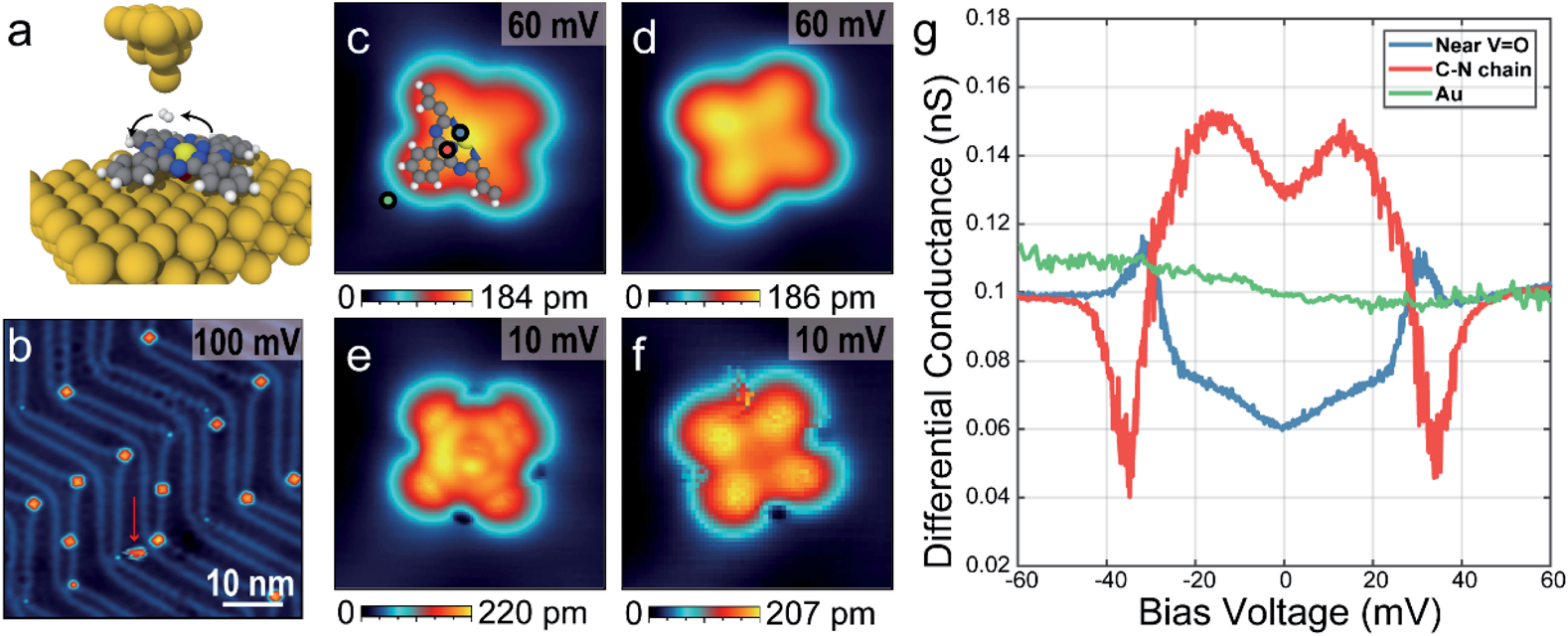
STM and spectroscopic measurements of a single VOPC molecule on the Au(111) surface. (a) A graphical representation of a single VOPc molecule on the Au(111) with hopping H_2_. V=O (light yellow) is located at the center normal to molecular plane and colors of balls with blue, grey and white represent nitrogen, carbon and hydrogen, respectively. (b) An STM topographic image of absorbed VOPc molecules on reconstructed Au(111) surface with *I*_set_ = 30 pA. Red arrow indicates a rotating molecule while scanning. (c–f) Single molecular STM images above (c and d) and below (e and f) the threshold voltage described in main text with O-up (c and e) and O-down (d and f) configurations. Images size is 3 nm × 3 nm. (g) d*I*/d*V* spectra near the center (blue), the C–N chain (red), and outside (green) of the VOPc molecule. The locations are indicated as coloured dots in (c).

The experiment was conducted on a home-built ultra-high vacuum STM/AFM at the base temperature of 8.5 K. AFM measurements were performed using a *q*-plus based force sensor (*Q* ∼ 10 000) with electrically isolated metallic tip, enabling simultaneous STM measurements. The VOPc molecule has a non-planar structure with the vanadyl (VO) center normal to the molecular plane surrounded by a C–N ring and four benzenes attached outside as shown in Fig. 1a. Fig. 1b shows the STM topographic image after depositing VOPc molecules on Au(111) surface. Most of the VOPc molecules are absorbed on the herringbone kink sites and exhibit two different appearances, which can be identified as O-up and O-down with equal probability. Molecules not adsorbed on the herringbone kink sites are easily rotated and moved during the scanning, for example as a molecule indicated by red arrow in Fig. 1b. Some molecules on the herringbone kink sites are still rotatable with high bias or high current indicating that the physisorbed molecules can have multiple rotational configurations with very low energy barrier in between. Both configurations show cross-shape features, similar to STM results of other metal-phthalocyanine molecules, but they show different contrast at the center.^16–18^ Magnified STM images for single molecules using 60 mV bias voltage as shown in Fig. 1c and d show that the center is brighter (darker) than the lobes in O-up (O-down) configuration. Moreover, in the O-down configuration, the molecules are tilted by about 3° in the molecular plane to the substrate. At the positive high bias voltage (*V*_bias_ = 2.4 V), the STM topographic image shows that the O-down molecules are brighter at the center than the O-up ones, because of the large portion of vanadium density of states (see ESI Fig. S1†). By comparison between DFT calculation and d*I*/d*V* maps, the O-up and O-down configurations are clearly distinguishable (see ESI Fig. S2 and S3†).

In the presence of H_2_ molecules, STM topographic images at 10 mV bias voltage as shown in Fig. 1e and f show striking differences with STM images at the same molecules using 60 mV as in Fig. 1c and d, respectively. For an O-up molecule (Fig. 1e), the STM image shows a more detailed chemical structure exhibiting a bright carbon–nitrogen (C–N) chain around the center and affixed four benzene rings. On the other hand, the O-down molecule (Fig. 1f) shows increased contrast between the center and the lobes. The scanning tunnelling spectra as shown in Fig. 1g show the excitation of molecular hydrogen at different sites within the molecule, which are symmetric in positive and negative bias voltage. It is noted that intra-molecular structure resolution at low bias voltage in STM imaging and unconventional spectral line shapes disappear after pumping hydrogen molecules out of the vacuum chamber (see ESI Fig. S4 and S5†), strongly suggesting that they are indeed induced by the presence of molecular hydrogen in the junction. At the C–N chain, marked with a red dot in Fig. 1b, the tunnelling conductance is increased near the Fermi level with excitation energy ∼30 meV (M-shape). However, the tunnelling conductance near the VOPc center, marked with a blue dot in Fig. 1b, decreases near the Fermi level with almost the same excitation energy at the C–N chain (U-shape). On the outside of the VOPc molecule, these features could not be observed (green dot in Fig. 1b).

**Fig. 2:**
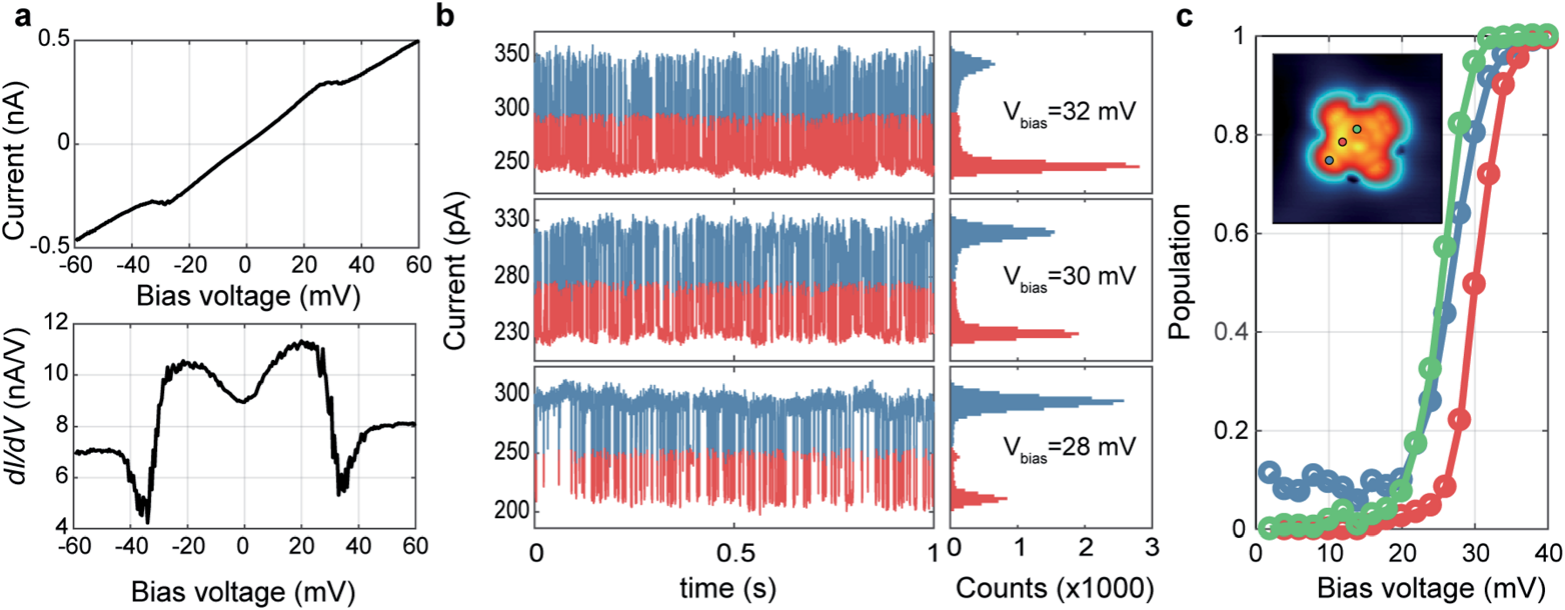
Random telegraphic noise analysis of the stochastic behavior of the trapped H_2_. (a) The bias dependent *I*–*V* (upper panel) and d*I*/d*V* (lower panel) spectra at N-site of a VOPc (marked by a red dot in (c)). (b) Time-dependent monitoring of the tunnelling current (left panels) and its histograms (right panels) measured at the same position in (a) with different bias voltages. The measurement is done under open feedback loop for 1 s with the sampling rate of 30 000 s^−1^. (c) The population probability of the excited state at different positions of VOPc. The measurement locations of each plot are indicated in the inset.

We investigated the spatial distribution of the stochastic behavior of molecular hydrogen by RTN measurements of the tunnelling current at various bias voltages near the threshold voltage. For these measurements, we recorded time-series of the tunnelling current data at varying bias voltages at a certain location after the tunnelling current feedback was opened at *V*_bias_ = 60 mV, *I*_set_ = 500 pA. Fig. 2a shows bias dependent tunnelling current and its differential conductance measurement at the location of one nitrogen (N) atom marked by a red arrow as shown in the inset of Fig. 2c. It shows clear conductance change and emerging noise around the transition voltage of about 30 mV. To investigate the noise source, we monitored the tunnelling current as a function of time around the transition voltage. Fig. 2b shows the RTN of tunnelling current and corresponding histograms at the same position as in Fig. 2a with bias voltages of *V*_bias_ = 32, 30 and 28 mV. RTN results clearly show there are two states with different tunnelling conductance that switch dynamically in an energy range of 20–35 mV. The histograms show two peaks representing the tunnelling current of these two states and the count of each bin is proportional to the probability of the system occupying the corresponding state at a given bias voltage. The population of the excited state can be calculated by the occupation time of the excited state divided by the total measurement time, *p* = *t*_excited_/*t*_total_. As shown in Fig. 2c, the population transition between two states occurs at *V*_bias_ = 30 mV at the N site. However, this threshold voltage is changed to 25 mV near the V=O center and the benzene ring, which are the same as the excitation energies in tunnelling spectra, respectively (see ESI Fig. S6†). Moreover, the residence time, defined as the averaged time of staying in one state before transitioning to the other state, is ∼1.5 ms measured at *V*_bias_ = 30 mV, where the probability of occupation of each state is 50%. This result is similar to previous STM measurements of H_2_ on Au(111) surface.^19^ Despite the different spectral shapes (M- and U-shape), we could observe the RTN at all the locations over the molecule in the same way. It is also noted that the conductance at a bias voltage larger than 50 mV becomes similar in all spectra to the outside of the molecule as in Fig. 1g and the same conductance was also recovered after removing H_2_ by pumping (see ESI Fig. S5†). This implies that the presence of the tip could always induce a local potential well and trap H_2_ between tip and VOPc. Near the threshold voltage, H_2_ fluctuates between these two states unless it is removed by a bias voltage higher than 50 mV.

The origin of these unconventional excitation spectra has been understood to be related to the quantum harmonic oscillator through an isotope-controlled experiment in a previous study.^14^ However, only a simple inelastic excitation of a quantum harmonic oscillator cannot explain the conductance drop, the huge dip or spike features of the d*I*/d*V* spectra, and the residence time scale of each conductance state. To explain all experimental observations, one needs to link a harmonic potential well to the two-state fluctuation. Our data suggest that there is a trapped state formed by the potential well that is induced by the tip and underlying VOPc molecule. When the bias voltage exceeds the threshold voltage, the trapped H_2_ will escape the potential well because the inelastic tunnelling process can give the trapped H_2_ enough energy to overcome the local potential barrier.

**Fig. 3:**
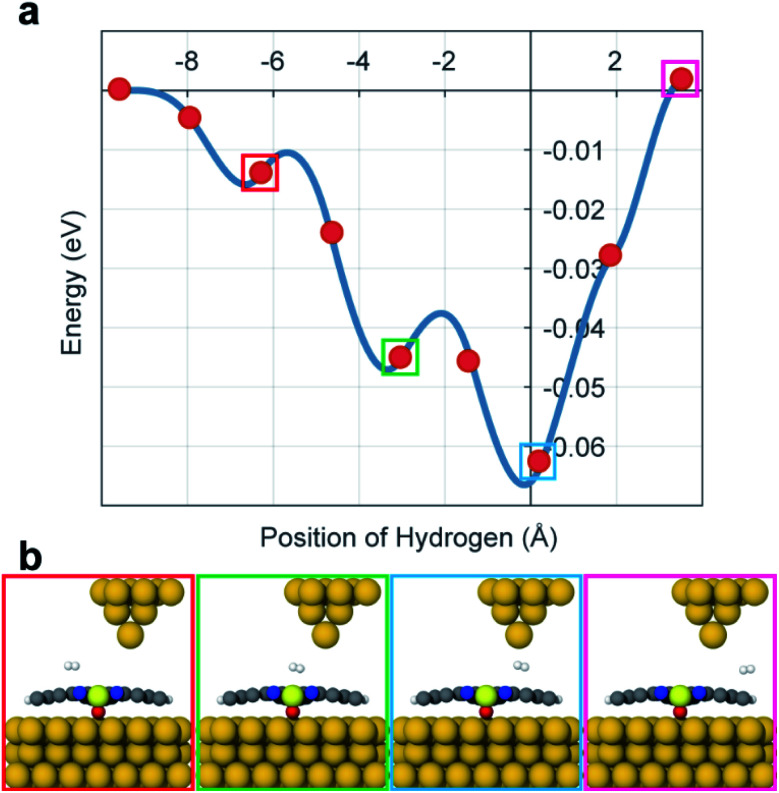
(a) The energy potential of H_2_ along the VOPc molecule. The distance is relative to the STM tip apex atom. (b) Graphical representations for selected points along the diffusion paths are shown.

To calculate the potential energy surface (PES) of H_2_ adsorbed on VOPc in the presence of the STM tip, we performed DFT calculations using plane-waves and pseudopotentials as implemented in quantum espresso (version 6.5).^20,21^ Details of the DFT calculations are described in the ESI (see ESI section 5).† Calculations indicate that the O-up configuration is energetically favorable compared to the O-down configuration by about 500 meV when using the Grimme-D3 Van der Waals correction.^21^ The deviating experimental observations indicate that the kink-site and kinetic effects during deposition might play an important role, resulting in O-up and O-down configurations with roughly the same probability. In the relaxed configuration, the V atom of an O-down (O-up) configuration sits roughly 350 (470) pm above the Au(111) surface and the organic ligand relaxes towards the gold surface in both cases. To mimic the STM tip, a pyramid shaped cluster of gold was added above the molecule. Surface diffusion of H_2_ across the molecule surface and below the tip was simulated by a nudged elastic band calculation along the long axis of the molecule which is taken as the *x*-axis here. Fig. 3a shows the PES that is created by the VOPc and the tip as a function of the lateral H_2_ distance from the tip and Fig. 3b shows the molecular configurations of selected locations of H_2_. The PES shows that the nitrogen positions on VOPc are local minima and in combination with the tip potential represents the global minimum along the chosen path. Moreover, the stronger interaction with N and V=O sites creates local minima near the global minimum with barrier heights that are in reasonable agreement with the threshold voltages measured in STM. The most plausible explanation of the observed two-state conductance in light of the calculated PES data is: (1) H_2_ is trapped under the tip below the threshold bias voltage. (2) At the threshold bias voltage, H_2_ starts to oscillate between the global minimum and a nearby local minimum. As the bias voltage increases, the possibility of residing in a nearby minima becomes higher. (3) When the bias voltage exceeds the tip-induced potential barrier, H_2_ diffuses away from the junction which resets the junction conduction to the conductance value without a H_2_ molecule. Since the exact reaction path of H_2_ under experimental conditions is unknown this “lateral shuffling” of H_2_ observed in the calculation is only one possible motion but alternative mechanisms cannot be excluded.

**Fig. 4:**
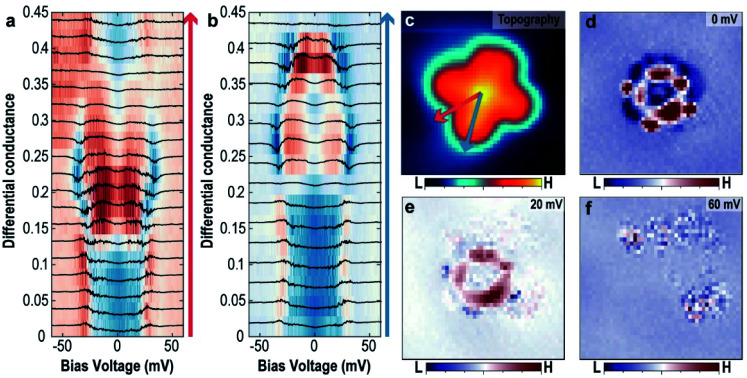
The spatial variation of the tunnelling spectra of the trapped H_2_ (a and b) evolution of the tunnelling spectra along the high symmetric lines of the VOPc molecule following red arrow (a) and blue arrow (b) as shown in (c). (c–f) Simultaneously obtained topographic image of the single O-up VOPc molecule (c) and d*I*/d*V* maps (d–f) with different bias voltages. Images size is 3 nm × 3 nm.

To further support this hypothesis, the variation of the excitation energy of the trapped H_2_ by the intermolecular interaction between H_2_ and VOPc molecules obtained from the tunnelling spectra at different locations within the molecule was measured. Fig. 4a and b show the evolution of the tunnelling spectra along the two symmetric lines of the VOPc molecule as shown in Fig. 4c. Following the blue arrow from the center to a benzene ring, the shape of the tunnelling spectrum changes from a U-shape to an M-shape at the N site. Beyond that, the excitation energy decreases from 30 meV to 20 meV when approaching the edge of the lobe. Following the red arrow in Fig. 4c, the spectra also change from a U-shape to an M-shape, but outside the VOPc molecule, the spectra change back to a U-shape. All U-shape spectra have in common that they are located near an oxygen atom or between two benzene rings. Further measurements of tunnelling conductance maps with different energies, as shown in Fig. 4d–f, show spatial variation of the tunnelling conductance, which implies a change of the interaction strength of the hydrogen involved in the tunnelling process. At *V*_bias_ = 0 mV (Fig. 4d), the map shows great contrast between U-shape (blue region) and M-shape (red region). The ring shape at the C–N chain around the V=O center and the four small affixed benzene rings are distinctive. At *V*_bias_ = 20 mV (Fig. 4e), the C–N chain still shows high differential tunnelling conductance but the benzene rings disappear. This is because the excitation energy at the benzene rings is smaller than the C–N chain and the bias voltage (20 mV) is almost at the threshold voltage at the benzene rings.

In summary, H_2_ trapped between tip and VOPc modifies the tunnelling conductance at low bias voltage by increasing the conductance near the C–N ring and decreasing it near the center and lobes. This is achieved by the hybridization of H_2_ with VOPc leading to a modified density of states.^14^ The threshold energy measured at different tip positions can vary since it depends on the barrier height between global and local minima, which further makes it dependent on the tip position. For example, the center and N sites show the largest excitation energy because the local minimum of the intermolecular interaction between H_2_ and VOPc is enhanced by the tip induced potential and results in the highest barrier towards a nearby local minimum. The tip height dependent tunnelling spectra show that the excitation energy increases when the tip gets closer to the VOPc molecule (see ESI Fig. S7†). This is again consistent with the picture of a tip-induced potential well that is stronger when the tip is in closer proximity. If the bias voltage is higher than the maximum threshold energy (>40 meV), there is no clear spatial variation in the tunnelling conductance as shown in Fig. 4f, indicating the absence of H_2_ at the junction.

**Fig. 5:**
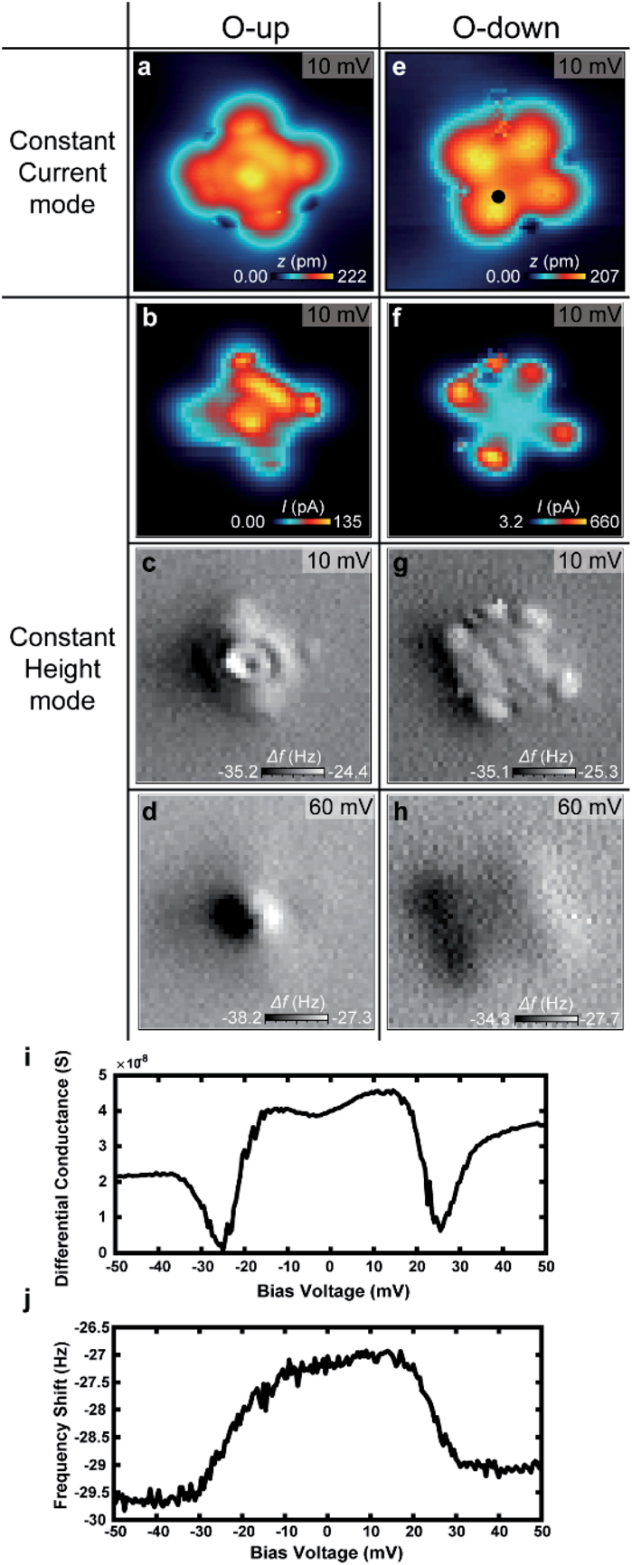
STM topographic images and the constant height mode tunnelling current and the frequency shift maps for O-up configuration (a–d) and O-down configuration (e and f) with trapped H_2_. (a and e) Constant current STM topographic images. (b and f) Tunnelling current map in constant height mode. (c and g) Frequency shift maps taken simultaneously with (b and f). (d and h) Frequency shift maps at 60 meV. Images size is 3 nm × 3 nm for all. The bias dependent (i) differential conductance and (j) frequency shift measured at the marked position in (b).

To verify the behavior of H_2_ trapped between the tip and VOPc, we used NC-AFM to measure the frequency shift (Δ*f*) at different positions of the VOPc. Fig. 5a–h show constant height maps of tunnelling current and Δ*f* for the two different molecular configurations (O-up and down), as confirmed by constant current images as shown in Fig. 5a and b. On VOPc molecules, Δ*f* maps show clear differences in Fig. 5c–h. Technically, the up-shift of Δ*f* means the introduction of repulsive force to the *q*-plus sensor.^22^ Δ*f* maps at a bias voltage with H_2_ trapped as shown in Fig. 5c and g show an up-shift of Δ*f* on VOPc due to the repulsive interaction between the tip and the trapped H_2_. For O-up molecules, the Δ*f* map in Fig. 5c shows the exact atomic position of oxygen as a dark spot at the center compared with the constant height current map in Fig. 5b. For O-down molecules, Fig. 5g shows higher spatial resolution than the tunnelling current map of Fig. 5f. Near the center of the molecule, the Δ*f* map shows a planar, two-fold symmetric orbital structure in contrast to the tunnelling current map. This implies that the Δ*f* signal is more sensitive to the details of the molecular conformation of H_2_ such as distance and angle from the tip at different tip positions. The combination of the bias dependent differential conductance and frequency shift in Fig. 5i and j tells us that not only electronic conductance but also mechanical force is introduced by the trapped hydrogen. The frequency shift shows clear up-shift below the threshold voltage, which also verifies the trapped hydrogen has repulsive interaction with the sensor. The presence of H_2_ in the junction enables ultra-high resolution imaging of the 2D chemical structure of the non-planar molecule VOPc. In the bias regime above 40 mV, we cannot observe the signature of hydrogen in the frequency shift maps shown in Fig. 5d and h. This indicates that the hydrogen is displaced from the tunnelling junction above the threshold voltage as discussed above.

## Conclusions

In conclusion, we have investigated the atomic-scale interaction between molecular hydrogen and a single VOPc molecule by using STM and NC-AFM. Our study revealed that the trapping mechanism of H_2_ is determined by the intermolecular interaction between H_2_ and VOPc on the atomic scale and further depends on the local tip-induced potential. Using DFT calculations, we could model the local potential energy landscape and we found potential energy barriers with qualitative and quantitative agreement with the experimental excitation energy measurements. Near the excitation energy, competition between the trapped state and the diffusion state explains the origin of two-state fluctuations in the tunnelling current. Furthermore, we could show that trapped H_2_ can be utilized for high-resolution imaging in NC-AFM. Further investigation of molecular interactions of H_2_ with different molecules composed of various atomic species would help to verify the interactions of H_2_ with molecules in more details. The understanding of molecular interaction of hydrogens with various molecules at the atomic scale will be able to provide more insight into improved molecular architecture of hydrogen related technologies.

## Conflicts of interest

There are no conflicts to declare.

## Supplementary Material

RA-011-D0RA08951F-s001
