# Supplementary material for: Atomic-scale intermolecular interaction of hydrogen with a single VOPc molecule on the Au(111) surface
Source: RSC Adv. 2021 Feb 3;11(11):6240–5. doi: 10.1039/d0ra08951f (PMC8694828; doi:10.1039/d0ra08951f)
Supplement: RA-011-D0RA08951F-s001 [file RA-011-D0RA08951F-s001.pdf]

Electronic Supplementary Material (ESI) for RSC Advances.
This journal is © The Royal Society of Chemistry 2021

Supporting information

Atomic-scale intermolecular interaction of hydrogen with a single VOPc molecule on the Au (111) surface

Jinoh Jung, [†a,b] Shinjae Nam, [†b,c] Christoph Wolf,[b,d] Andreas Heinrich[*b,c], and Jungseok Chae[*b,d]

a. Department of Physics, KAIST, Daejeon 34141, Korea
b. Center for Quantum Nanoscience, Institute for Basic Science (IBS), Seoul 03760, Korea.
c. Physics Department, Ewha Womans University, Seoul 03760, Korea
d. Ewha Womans University, Seoul 03760, Korea

† These authors contribute equally
** Corresponding authors; AJH: heinrich.andreas@qns.science,*
JC: chae.jungseok@qns.science

1. Absorption configurations of VOPc on the Au (111) surface

There are two different absorption configurations of VOPc on the Au (111) surface. The oxygen atom of the VOPc points toward the vacuum (O-up) or the surface (O-down) when it is absorbed on the substrate. The two types of absorption configurations are clearly distinguished in Fig S1. (a) and (b). We measured the same region with different bias voltages, 0.1 V and 2.4 V. At V_{bias}=0.1 V, we could see the image of the molecule's geometric shape because the density of states (DOS) of VOPc hybridized with underlying Au surface is dominant and DOS of VOPc is relatively small. At V_{bias}=2.4 V, the DOS of vanadium at the center of the O-down molecule is so large that the O-down configuration shows much brighter.

The O-down molecule on the Au (111) surface was calculated by DFT calculation as shown in Fig. S2a. In O-down case, the DOS of the vanadium *d* orbital is dominant near 1.5 eV and Fermi level. Compared to the *dI/dV* maps in Fig. S2b measured at different voltages of 0.4 V and 1.5 V, the O-down molecule shows higher *dI/dV* signals which is proportional to the DOS at the center compared to the O-up molecule. In the O-up molecule, there is no obvious difference in the *dI/dV* maps at the center from the lobes on the molecule under the same conditions. The reason for the higher signal at the center of the O-down molecule in topographic images and *dI/dV* maps is because the center vanadium atom is closer to the tip, so the orbital overlap between the tip and the vanadium atom is larger than the O-up one.

We can identify O-up and O-down molecules at V_{bias}=0.1 V based on STM images in Fig. S3 calculated from DFT using the Tersoff-Hamann approximation.[1]

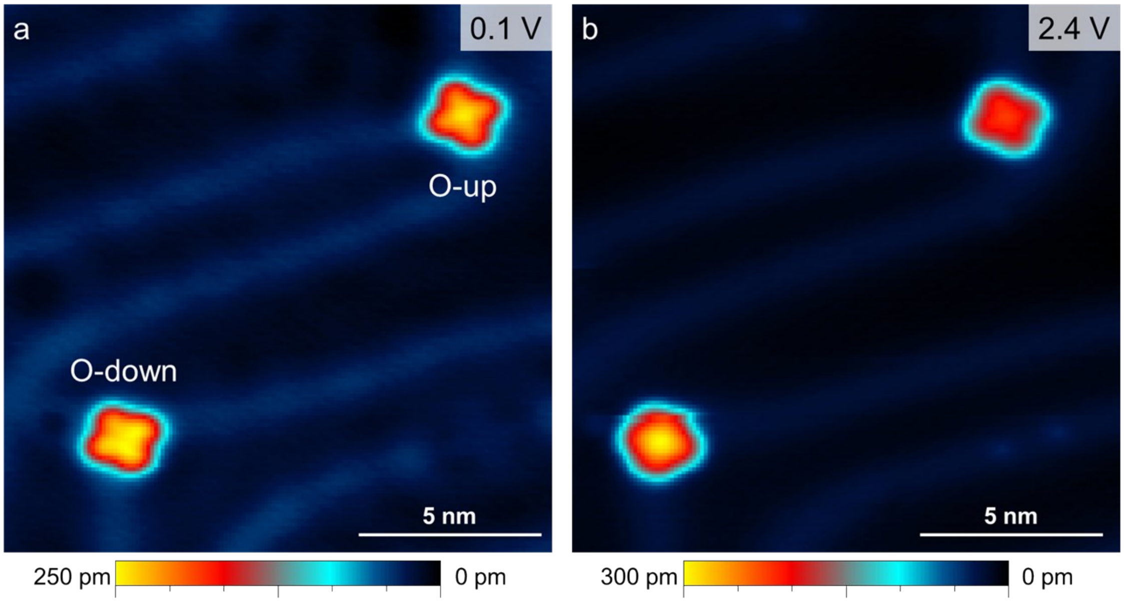

Figure S1. Bias dependent topographic images of O-up and O-down configurations of VOPc on the Au (111) surface. (a) V_{bias}=0.1 V and (b) V_{bias}=2.4 V

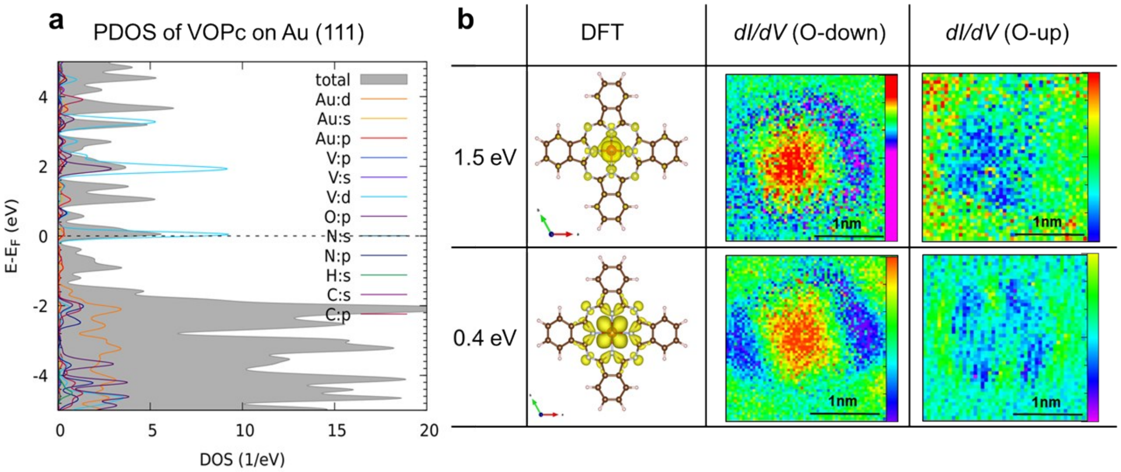

Figure S2. (a) The projected density of states of the O-down VOPc on the Au (111) surface. (b) Spatial distribution of calculated density of states and measured *dI/dV* maps for O-down and O-up configurations of single VOPc molecules.

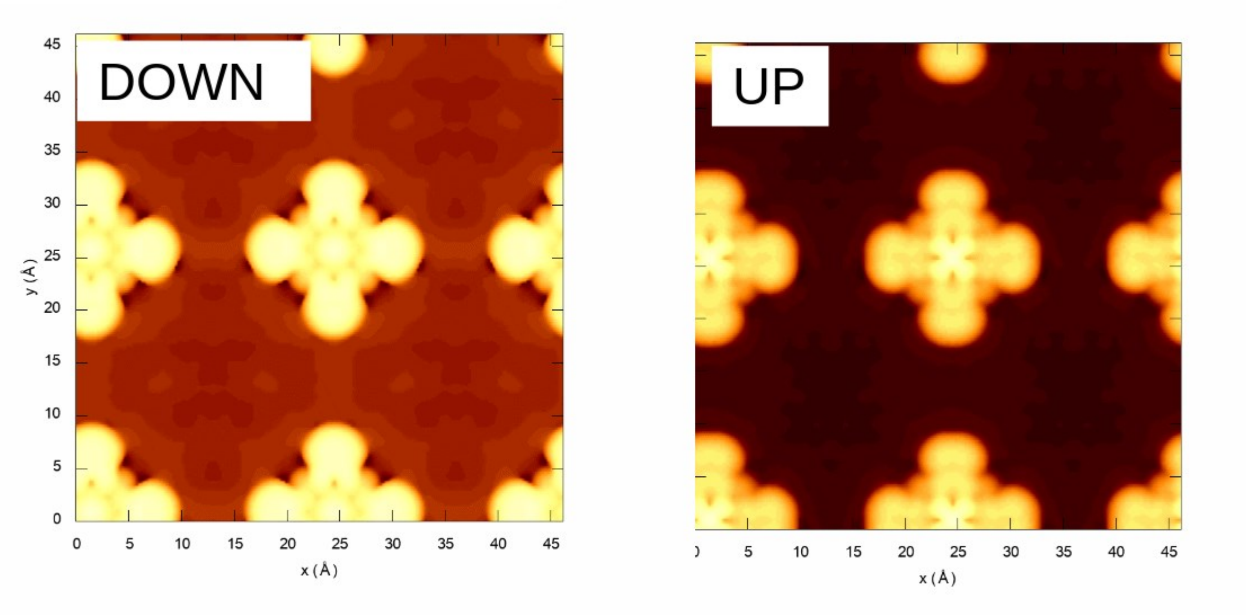

Figure S3. STM images for O-down and O-up at 100 mV bias clearly showing the bright center in the case of the O-up configuration compared to the relatively bright ligands in the case of O-down.

2. Comparison of STM results between with and without molecular hydrogen

Accumulated H_2 on the sample can be removed by increasing the temperature of the sample around 40 K. During the heating process, we confirmed that the pressure increased from <1.0e-10 to 2.0e-9 torr while the doors for two shields open. After outgas was done, the pressure went back to the base pressure and then we cooled down the sample to the base temperature. After this process, we couldn't observe the evidence of H_2 at the STM images and the tunneling spectra at low bias as shown in Fig. S4 and S5.

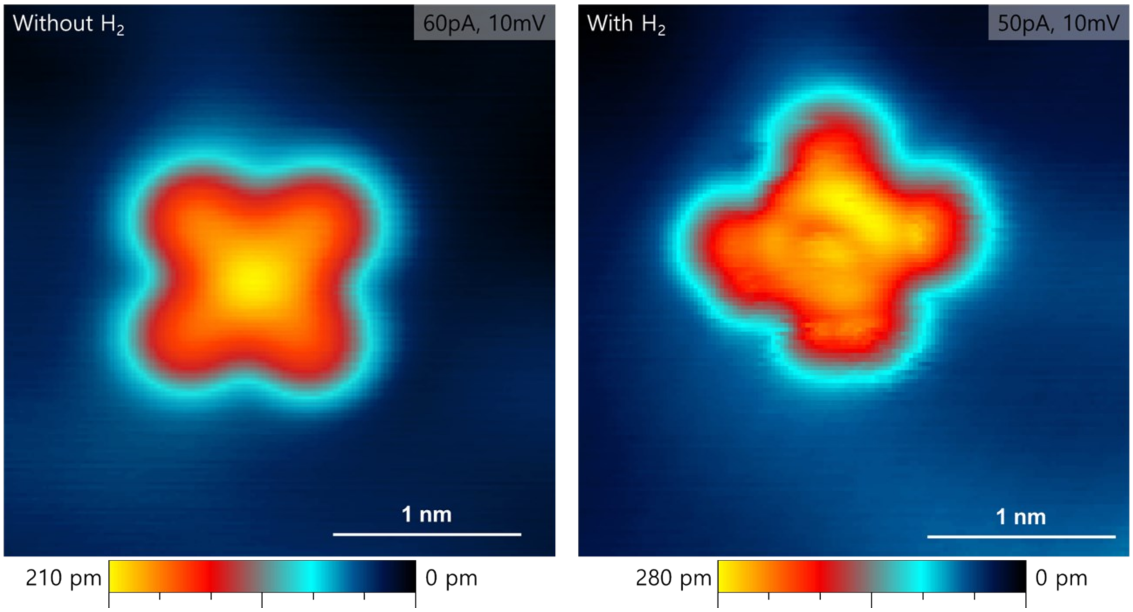

Figure S4. STM topographic images without / with H_2. (a) STM image at the bias voltage of 10 mV without H_2. (b) STM image with H_2 using the same bias voltage of 10 mV.

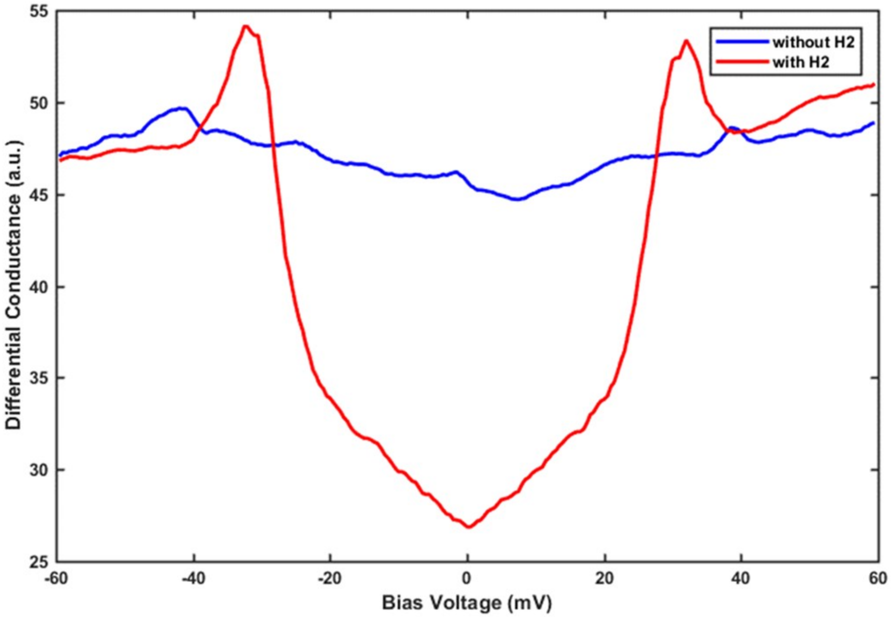

Figure S5. *dI/dV* spectra near the center of VOPc without / with H_2. The spectra are normalized by the setpoint current. The setpoint is 550 pA for red and 275 pA for blue.

3. Tunneling spectra at RTN measurement positions.

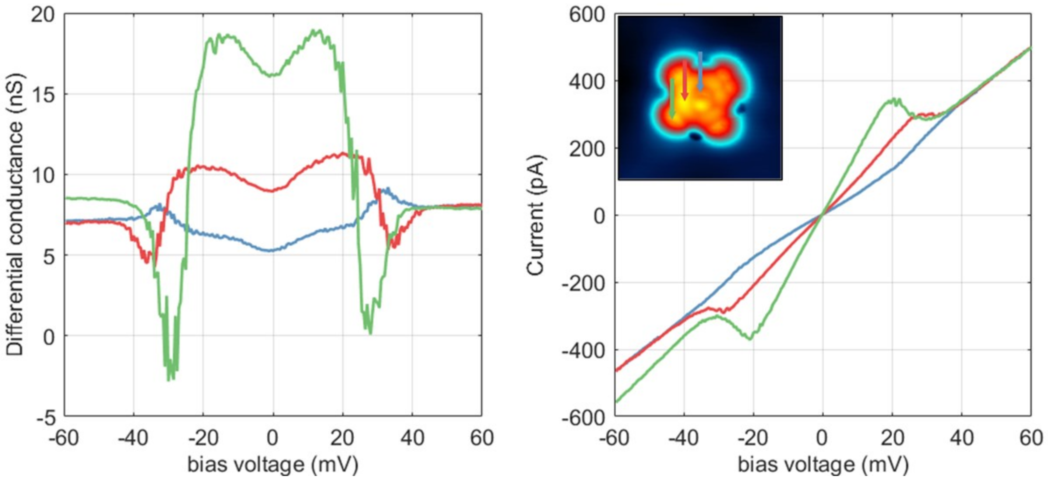

Figure S6. *dI/dV* and I-V spectra at the same positions of the RTN measurements marked in Fig. 2c.

4. Tip height dependent tunneling spectra

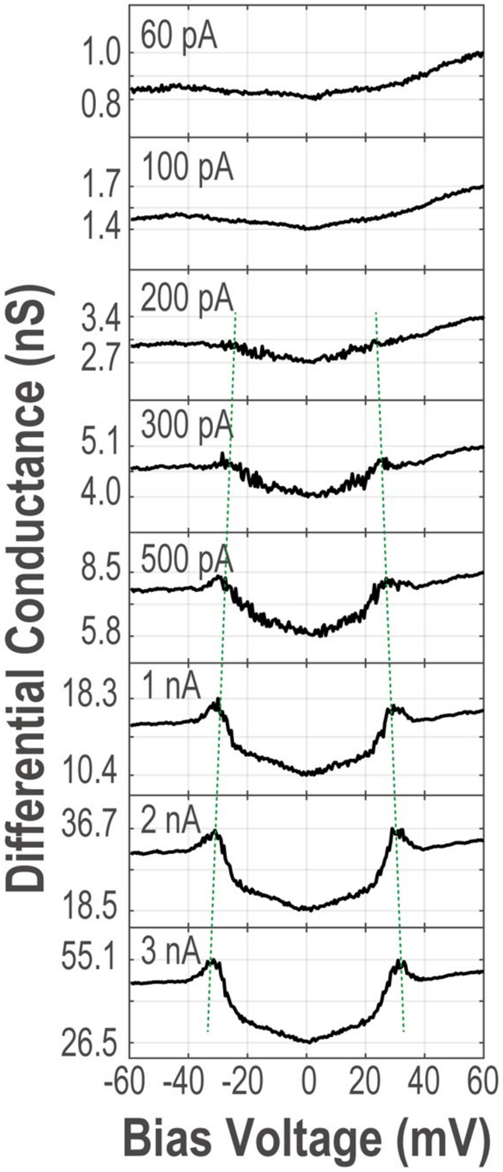

Figure S7. Tip-height dependent tunneling spectra near the center of the VOPc molecule. The setpoint current is indicated at upper-left corner. Green dotted line is a guide line along the positions of conductance spike for each spectrum.

5. Additional DFT calculations

Pseudopotentials were chosen according to recommendations from the SSSP efficiency set with cutoffs for wave functions and charge density as indicated, except for the gold pseudopotentials which was taken from the GBRV library.[2,3] We tested the convergence with an in-plane k-point grid up to 3x3 but found no difference compared to calculations only including the gamma point. For subsequent calculations only the gamma point was used. The system was modelled as 3 monolayers of gold exposing the (111) facet expanded in a lateral supercell of about 20x20 Angstroms and padded by 20 Angstrom of vacuum in z-direction. VOPc was added and the system was relaxed with the Au bottom layer frozen until the forces were less than 10^{-3} Ry/a_0 (a_0 is the Bohr radius). In addition, to estimate the local potential induced by the tip we performed the same nudged elastic band calculations as discussed in the main text without the tip. It clearly shows an

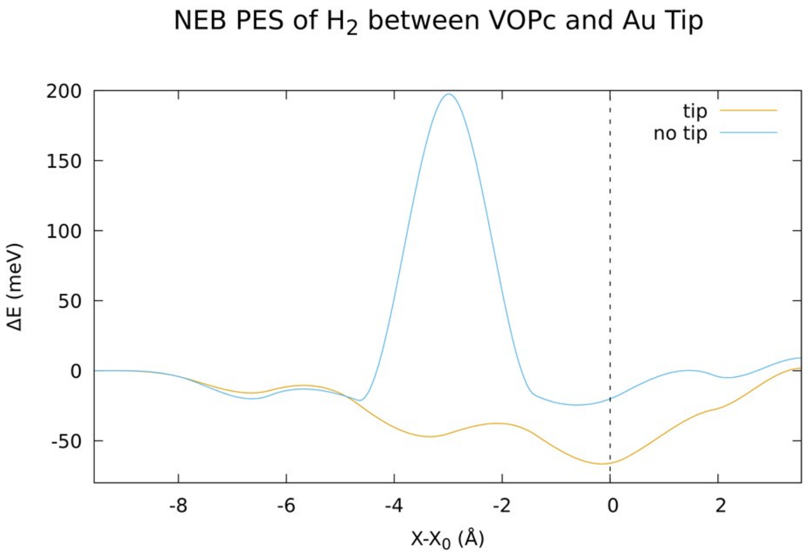

Figure S8. Difference in PES for lateral diffusion of H_2 with and without tip. X_0 marks the location of the tip apex.

attractive potential for the H_2 molecule near the nitrogen atoms and a strongly repulsive region near the V-O core.

REFERENCE

(1) Tersoff, J.; Hamann, D. R. Theory and Application for the Scanning Tunneling Microscope. *Phys. Rev. Lett.* **1983**, *50* (25), 1998–2001. https://doi.org/10.1103/PhysRevLett.50.1998.

(2) Garrity, K. F.; Bennett, J. W.; Rabe, K. M.; Vanderbilt, D. Pseudopotentials for High-Throughput DFT Calculations. *Comput. Mater. Sci.* **2014**, *81*, 446–452. https://doi.org/10.1016/j.commatsci.2013.08.053.

(3) Prandini, G.; Marrazzo, A.; Castelli, I. E.; Mounet, N.; Marzari, N. Precision and Efficiency in Solid-State Pseudopotential Calculations. *npj Comput. Mater.* **2018**, *4* (1). https://doi.org/10.1038/s41524-018-0127-2.
